# Head circumference assessment in pediatric MRI: a pilot study of manual measurement methods and automated segmentation-based alternatives

**DOI:** 10.3389/fnins.2026.1822095

**Published:** 2026-06-12

**Authors:** Jan Philipp Adam, Loukia M. Spineli, Hinrich Boy Winther, Eva Bültmann

**Affiliations:** 1 Institute of Diagnostic and Interventional Neuroradiology, Hannover Medical School, Hanover, Germany; 2 Midwifery Research and Education Unit, Hannover Medical School, Hanover, Germany; 3 Institute for Diagnostic and Interventional Radiology, Hannover Medical School, Hanover, Germany

**Keywords:** automatic biometry, automatic segmentation, head circumference, magnetic resonance imaging, neuropediatrics

## Abstract

**Purpose:**

Head circumference (HC) is an important clinical parameter in neuropediatrics, but it is often missing or outdated in referral information. This can lead to subjective, reader-dependent estimation during MRI interpretation. We first aimed to compare magnetic resonance imaging (MRI)-based methods for HC measurement against the tape measure (ground truth), and second to establish an automated alternative.

**Methods:**

In 23 children (mean age 4.5 years, range 0.5–17 years), HC was prospectively measured with a tape measure (ground truth) on the day of MRI. MRI-based HC measurements were derived from 3D T1-weighted MPRAGE and followed a two-step workflow: measurement plane selection and circumference measurement within that plane. Plane selection was performed using visual-based, rule-based, atlas-based [(infant) FreeSurfer], or neural network (nn)-based methods. Circumference measurement was performed using manual ellipsoid, manual contour, automated ellipsoid, or automated contour methods. The relative technical error of measurement (r-TEM; acceptable < 1.5%) and intraclass correlation coefficient (ICC; two-way mixed ANOVA model) were used to assess accuracy and consistency with the tape measure.

**Results:**

Visual-based with manual ellipsoid/contour and rule-based with manual ellipsoid/contour showed acceptable accuracy (r-TEM 0.73%–1.12%). Visual-based with automated ellipsoid and rule-based with automated ellipsoid also demonstrated acceptable accuracy (r-TEM 0.77% and 0.68%). Atlas-based with automated ellipsoid achieved the lowest r-TEM (0.55%), followed by nn-based with automated ellipsoid (r-TEM 0.75%). In contrast, automated contour approaches showed unacceptable accuracy (r-TEM 3.42%–4.21%). Seven nn-based measurements with automated ellipsoid/contour were spurious. ICCs were high across all methods (0.993–0.997); however, manual contour and automated ellipsoid were associated with overfitting issues.

**Conclusion:**

The developed, fully automated algorithm based on (infant) FreeSurfer provides precise and reliable head circumference measurements from pediatric MRI scans with acceptable overall accuracy and excellent consistency with manual measurements using a tape (gold standard). Our algorithm simplifies the head circumference measurement process and provides a reproducible, reader-independent value that enhances the interpretation of neuroradiological findings. Further studies should be conducted to validate with larger sample sizes and to develop deep neural network algorithms for segmentation.

## Introduction

Head circumference (HC) is an essential clinical parameter that correlates with intracranial volume, brain development, and neurodevelopmental outcomes (Bray et al., 1969; Watemberg et al., 2002; Harris, 2015; Leong et al., 2021). Serial evaluation of HC is integrated into routine pediatric examinations by measuring the frontooccipital circumference with a non-stretchable tape and comparing the results with extensive data from age-specific national surveys (Stolzenberg et al., 2007; Schienkiewitz et al., 2011). A circumference below two standard deviations or the 3rd percentile is defined as microcephaly, while above two standard deviations or the 97th percentile is defined as macrocephaly (Winden et al., 2015; Kaindl, 2017). Macrocephaly is often accompanied by cerebrospinal fluid disorders (CSF), such as hydrocephalus caused by aqueduct stenosis or congenital/tumorous malformations, but rare metabolic disorders are also associated (Orrù et al., 2018). Typical differential diagnoses of microcephaly include structural brain disorders and infectious, toxic, or metabolic agents, in addition to genetic syndromes (Passemard et al., 2013). Early identification of pathologic head circumference is crucial for investigating disorders and often leads to further evaluation with magnetic resonance imaging (MRI) (Ashwal et al., 2009).

Unfortunately, when reading pediatric MRI studies, HC values are not always reported by attending physicians or are outdated. Poor clinical data and sometimes missing diagnostic hypotheses can lead to discrete changes being overlooked. Radiologists must rely on their visual impressions using the craniofacial ratio as a surrogate parameter or measure an approximate image-based circumference by themselves–both are subjective approaches. Additionally, the human error rate in manual tape measurements should also not be underestimated.

Only a few studies have addressed the estimation of head circumference from MRI and Computed Tomography (CT) images, yet they still have limitations. These studies have primarily been designed retrospectively, and agreement was assessed using clinical measurement validation data collected up to 25 days (Mourão et al., 2023) or 3 months (Rau et al., 2021) apart from the CT/MRI scan. Furthermore, subjective factors remain, as the reader chooses the optimal axial plane with the supraorbital bulge as the anterior starting point for frontooccipital head circumference, rather than a rule-based method determining it. This subjective assessment can be challenging and not reproducible due to abnormal head shapes, which are particularly common in children with abnormal head circumference. In addition, previous studies have shown inconsistent results with the circumferential approach. Some studies used an ellipsoid region of interest (ROI) for easier handling (Rau et al., 2021; Yepes-Calderon et al., 2021). In contrast, others utilized tools designed to measure geometric skull shapes to approximate the gold standard tape measurement as closely as possible (Mourão et al., 2023). Moreover, perfectly round shapes have also been employed to develop an automated measurement tool for HC (Yepes-Calderon et al., 2021).

Subjective and reader-dependent measurements combined with incomplete clinical data highlight the need for a new, simplified, reproducible MRI-based method of measuring pediatric head circumference. A fully automated MRI-based measuring algorithm could lead to an objective and rapid analysis, minimizing human susceptibility to errors. Therefore, this study was designed as a pilot project to indicate the best MRI-based manual method and to establish a reliable, fully automated MRI-based measuring algorithm.

## Materials and methods

This prospective study was approved by the local ethics committee, as all parents provided written informed consent for clinical research. Guidelines for reporting reliability and agreement studies (Kottner et al., 2011) as well as the principles of the Declaration of Helsinki, were respected.

### Patients

We prospectively included 23 pediatric patients (mean age 4.5 years, range 0.5–17 years, 9 female and 14 male) undergoing a brain MRI under anesthesia at the Medical School Hanover from January to March 2024. The brain MRI was performed for various clinical indications, including developmental delay, deafness, hydrocephalus, intracranial hemorrhage, and tumor staging. Our standard brain protocol included a sagittal 3D-T1w MPRAGE (Magnetization Prepared RApid Gradient Echo) acquired using different MRI scanners (Siemens 1,5T Magnetom Aera and Avanto as well as 3T Magnetom Verio and Skyra, Siemens Healthineers, Erlangen, Germany) with the following parameters: TR 2200/1980/1800/2300 ms, TE 2.67/3.98/2.27/2.32 ms, flip angle 8°/15°/9°/8° (Aera/Avanto/Verio/Skyra), and 1 mm slice thickness. Signal abnormalities caused by shunt devices and motion artifacts were not reasons for exclusion, as we aimed to validate real-life conditions.

### Calibration data

A separate set of 10 clinical MRI scans of children aged between 0.5 and 9.3 years (6 female and 4 male) was used as a calibration set for the development of an algorithm for the automatic detection and alignment of the optimal axial measurement plane.

### Tape measurement (ground truth)

Prior to the MRI scan, an experienced neuroradiologist (E.B.) measured the children’s head circumference using a non-elastic tape recognized as the gold standard. According to institutional standards, the tape was wrapped around the head supra-auricularly, between the supraorbital bulge anteriorly and the occipital protuberance posteriorly.

### Manual MRI-based measurement

**FIGURE 1:**
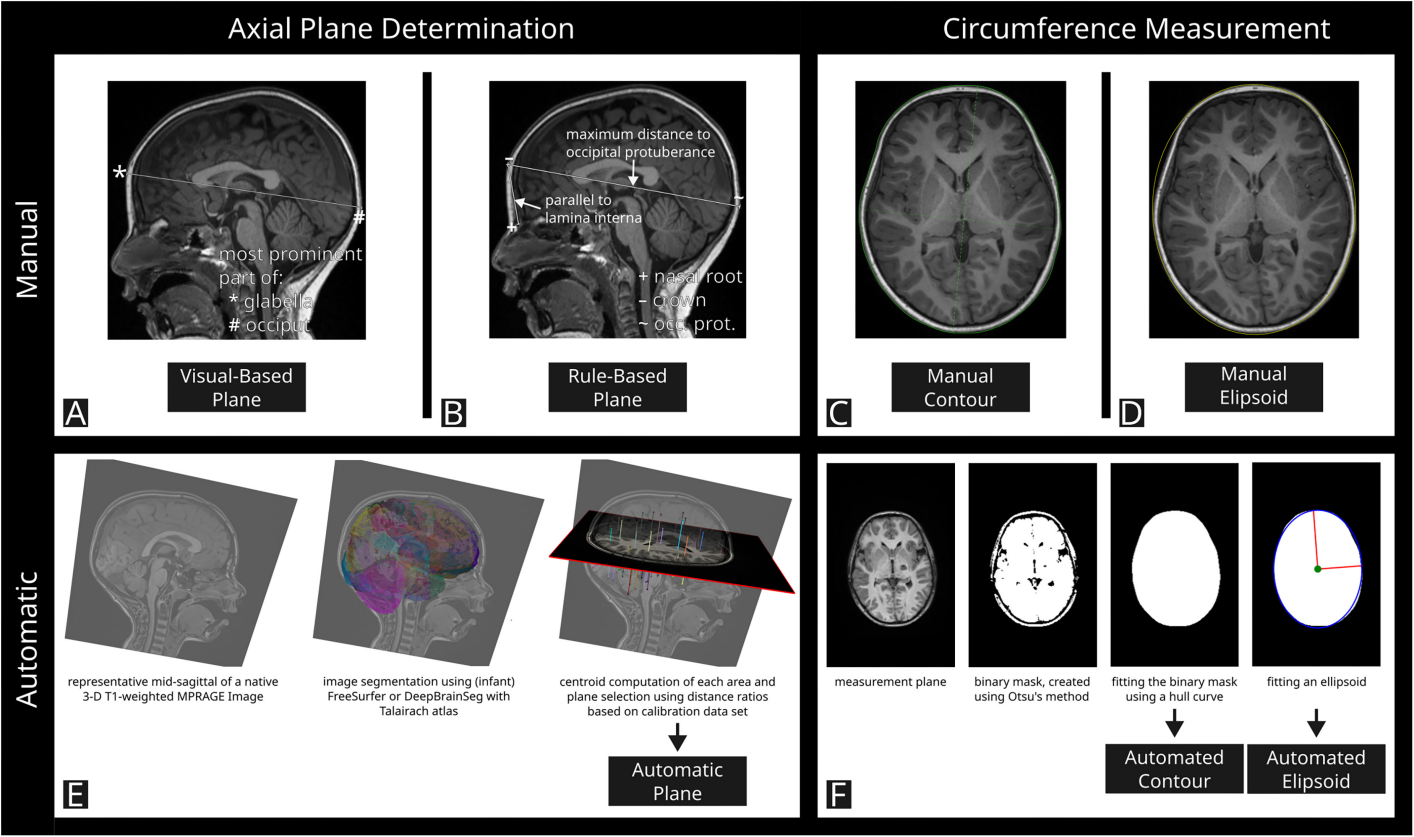
The figure is divided into four main panes, separating the manual and automated approaches as well as determining the axial measurement plane and the method for performing the measurement. **(A)** Depicts how we determined the measurement plane visually, **(B)** demonstrates the rule-based manual approach, in contrast to panel **(E)**, which employs a fully automated method. **(C,D)** Depict the manual contour **(C)** and ellipsoid **(D)** approach (please note the fine contour lines), in contrast to panel **(F)**, which depicts the fully automated determination of the circumference and ellipsoid.

Image analysis was performed by a 5th-year radiology resident experienced in adult and pediatric neuroimaging using the in-house Picture Archiving and Communicating System (PACS) (Visage 7.1, Visage Imaging Inc., San Diego, CA, USA). The reader was blinded to patient age, clinical data, and prior tape measurements. Manual head circumference measurement was performed in the axial plane by first placing an ellipsoid region of interest (ROI) and, secondly, a manual (freehand) ROI contour around the scalp, mimicking the tape measurement (Figures 1C,D). Before measuring, the axial plane needed corrections in the three anatomical dimensions: coronal slices aligned with the Margo supraorbitalis and sagittal slices parallel to the falx cerebri. The axial plane selection was performed in two separate ways: for easy handling, the reader defined the optimal axial plane subjectively, starting from the most prominent part of the glabella to the most prominent part of the occiput (visual-based method) (Figure 1A). For a standardized approach, we drew a line in the corrected sagittal plane starting from the nasal root to the crown parallel to the lamina interna. The intersection with the cutis was defined as the anterior starting point for the axial plane and covering the maximum distance to the occipital protuberance (rule-based method) (Figure 1B). This results in four manual measurements (visual- and rule-based plane selection with manual ellipsoid or contour head circumference measurements).

### Automating the process

The steps for the semi- and fully automated methods evaluated in this study were to first manually or automatically identify a measuring plane and then to perform an automated head circumference measurement. The aforementioned steps of the manual method for plane selection can be combined with the automated circumference measurements described below to create a semi-automated workflow, e.g., rule-based plane selection in combination with an automatically fitted ellipsoid. This results in four semi-automated methods (visual- and rule-based plane selection, with automated ellipsoid or contour). For the four fully automated measurements, the plane selection was also performed automatically using (infant) FreeSurfer or DeepBrainSeg, respectively (atlas- or nn-based plane selection with automated ellipsoid or contour head circumference measurement).

### Automation of measurement plane selection

We used FreeSurfer (Fischl, 2012) and infantFreeSurfer (infantFS) (Zöllei et al., 2020) to perform an atlas-based segmentation. These tools use anatomical mapping information to identify and delineate cranial structures in T1-weighted MRI scans. Because the performance of the standard (adult) FreeSurfer pipeline is known to decline in very young children, we applied an age-stratified strategy.

For subjects aged ≥ 4.5 years, brain segmentation and parcellation were performed with FreeSurfer using the standard recon-all stream, which includes intensity normalization, skull stripping, subcortical segmentation (aseg), and cortical surface/parcellation steps. The resulting volumetric label maps (e.g., aseg and cortical parcellations mapped into the segmentation volume) served as the basis for extracting stable anatomical reference points used by the automated plane estimation algorithm (atlas-based measurement plane selection).

For subjects under 4.5 years, segmentation was performed using infantFS, an automated FreeSurfer-compatible pipeline for infant brains, originally developed for T1-weighted MRI in the 0–2-years age range. The infantFS workflow was executed via the recommended command-line interface (infant_recon_all, with age provided in months) and produced FreeSurfer-style outputs (including aseg-style segmentations) suitable for the same downstream processing as in older children. We selected infantFS for younger subjects to better accommodate developmental anatomy and age-dependent tissue contrast, consistent with the FreeSurfer team’s recommendation to prefer the infant pipeline below ∼4.5 years.

Additionally, we tested the deep neural network DeepBrainSeg (Kori et al., 2018) (nn-based) for brain parcellation as a substitute of (infant) FreeSurfer. We wanted to explore the deep learning approach for image segmentation, as it yields great speed benefits.

Regardless of the method used for image segmentation (either atlas-based or nn-based), the next step was to develop an algorithm to determine the measurement plane from the parcellation. For this, a separate set of 10 pediatric MRI scans, previously annotated using the visual-based method, served as a reference for training the algorithm to automatically detect and align the optimal axial plane. The algorithm determined the center of mass for each segmented structure (area). The average spatial relationship between these centers of mass and the manually delineated axial slice for head circumference measurement is used to calibrate the algorithm. This spatial information is then used to estimate the optimal plane for the head circumference measurement plane within new scans.

### Automation of the head circumference measurement

With a given measurement plane, a binary “head/scalp” mask was created by intensity-based segmentation (foreground–background separation) using Otsu’s method (Otsu, 1979), followed by a morphological post-processing step to obtain a closed, contiguous region representing the outer head outline using the convex_hull_image function of scikit-image version 0.24.0. This also reduced sensitivity to local indentations or small discontinuities and provided a robust approximation of the external scalp boundary.

These boundaries were used to derive two types of circumference approximations: an automated ellipsoid region of interest (ROI), which represents a simplified geometrical estimate of the head shape, and the automated circumference contour.

### Statistical analysis

We used the relative technical error of measurement (r-TEM) proposed by Perini et al. (2026) to investigate the variation of metric measurements performed by different anthropometrists. We preferred r-TEM to other assessment methods of inter-measurement accuracy, such as Pearson’s correlation coefficient, intra-class correlation coefficient (ICC), and paired samples *t*-test, for having a comparatively better performance according to a recent simulation study by Fancourt and Stephan (2018). Lower r-TEM values indicate high accuracy and measurement quality. Perini et al. (2026) suggested using the 2.0% and 1.5% thresholds to indicate correspondingly acceptable r-TEM values for beginner and skilled researchers performing the measurements [Table 1 in Perini et al. (2026)]. Hence, r-TEM values exceeding these thresholds were perceived as reflecting unacceptable measurement errors, potentially reflecting low accuracy. The methodology as well as the thresholds were in line with previous studies investigating similar anthropometric measurements in cross-sectional imaging, so extrapolation in this approach strengthened consistency and comparability with the existing literature.

**TABLE 1:** Mean difference (bias) and standard deviation of different measurement methods with the tape.

Automation type	Measurement plane selection	Circumference type	Mean difference	Standard deviation
Manual	Visual-based	Manual ellipsoid	−2.71	5.66
Manual	Visual-based	Manual contour	0.97	5.31
Manual	Rule-based	Manual ellipsoid	−5.24	5.64
Manual	Rule-based	Manual contour	0.23	5.08
Semi-automated	Visual-based	Automated ellipsoid	0.18	5.38
Semi-automated	Visual-based	Automated contour	24.86	4.91
Semi-automated	Rule-based	Automated ellipsoid	0.18	4.78
Semi-automated	Rule-based	Automated contour	24.58	4.14
Fully-automated	Atlas-based	Automated ellipsoid	−0.38	3.82
Fully-automated	Atlas-based	Automated contour	23.65	4.28
Fully-automated^1^	nn-based	Automated ellipsoid	0.19	4.48
Fully-automated^1^	nn-based	Automated contour	24.69	4.48

nn, neural network; SE, standard error.

^1^Seven measurements were excluded from the nn-based approach because they were spurious.

We also performed two *ad hoc* analyses, as requested by the reviewers: (i) we calculated r-TEM separately for children up to 4.5 years old, and those older than 4.5 years, where the age cut-off of 4.5 years for switching between infantFreeSurfer and standard FreeSurfer coincided with the mean age of the cohort, meaning roughly half the patients were processed with each pipeline; and (ii) we calculated the mean difference of each method against the tape measurement, which indicates the bias, namely, the difference from the identity line.

Nevertheless, we also reported the ICC as a supplementary measure to the r-TEM, as it is a widely used reliability measure in the medical community. We calculated ICC using a two-way mixed effects ANOVA (Analysis of Variance), where children were the random effects and the different HC measuring approaches were the fixed effects, since we were interested in inferring only the approaches above. We accounted for possible systematic error between the different measurement methods and used a single rating to treat the tape measurement as the gold standard. Following Koo and Li (2016), we interpreted ICC above 0.90 as excellent consistency, between 0.75 and 0.90 as good consistency, between 0.50 and 0.749 as moderate, and below 0.50 as poor consistency. ICC was reported alongside a 95% confidence interval.

Note that both r-TEM and ICC assess reliability but target different aspects of the measurement error: r-TEM measures accuracy (measurements are close to the truth), whereas ICC measures consistency of the measurements. The measurements may be consistent (close to each other) but not accurate, and vice-versa. All analyses were performed using R Statistical Software (Version 4.3.3) (R Core Team, 2021) and Microsoft Excel 2016 (Microsoft Corporation, Redmond, WA, USA). The psych R package (William, 2024) was employed for the two-way mixed-effects ANOVA, and the ggplot2 R package (Hadley, 2016) was implemented to create the graph for the analysis.

## Results

### Sample

This study included 23 children: nine females (40%) and 14 males (60%). The children’s ages ranged from 0.5 to 17 years, with a mean age of 4.5 years. Head circumferences varied from approximately 38 to 54 cm across all tape, manual, and automated measurements. Based on tape measurement (the gold standard), four patients were identified as microcephalic (17.4%) - all of whom had severe microcephaly at or below the 1st percentile – and three patients had borderline measurements near pathological values (4th and 96th percentiles).

### Accuracy and consistency of manual, semi- and fully automated measurements

For the nn-based measurements with automated ellipsoid/contour pipeline (DeepBrainSeg), a subset of examinations had to be excluded after quality control because the underlying segmentations were not reliable enough to derive a valid scalp circumference. Specifically, seven scans showed gross segmentation failures.

Table 2 summarizes the r-TEMs from different measurements. Tape measurements had an acceptable accuracy with manual, MRI-based measurements [visual- or rule-based plane selection with manual head circumference measurement (ellipsoid or contour)], ranging from 0.73% to 1.12%. An acceptable accuracy was also found for visual-based and rule-based with automated ellipsoid (r-TEM = 0.77% and 0.68%, respectively), as well as for atlas-based and nn-based with automated ellipsoid (r-TEM = 0.55% and 0.75%). However, no acceptable accuracy with the tape measurement was found for visual-based and rule-based plane selection with automated contour (r-TEM = 3.61% and 3.55%, respectively), for atlas-based and for nn-based with automated contour (r-TEM = 3.42% and 4.21%, respectively). When distinguishing between children up to 4.5 years (*n* = 13) and older (*n* = 10), the conclusions regarding the accuracy of the different methods (as compared to the tape measurements) remain unaffected (Table 3). However, as compared to the whole sample (Table 2), r-TEM values from older children tend to be slightly lower and r-TEM values from younger children tend to be slightly larger. The unacceptable accuracy of all automated contour methods with the tape measurement was in line with the substantial average bias observed, ranging from 23.65 (atlas-based) to 24.86 (visual based) (Table 1). Namely, the measurements from the automated contour methods were on average larger than those by the tape measurement.

**TABLE 2:** Accuracy and consistency of different measurement methods with the tape.

Automation type	Measurement plane selection	Circumference type	r-TEM		ICC	
			Value	Acceptable?^1^	Value	Consistency
Manual	Visual-based	Manual ellipsoid	0.90%	Yes	0.993 (0.983, 0.997)	Excellent
Manual	Visual-based	Manual contour	0.77%	Yes	0.994^2^ (0.985, 0.997)	Excellent
Manual	Rule-based	Manual ellipsoid	1.12%	Yes	0.993 (0.983, 0.997)	Excellent
Manual	Rule-based	Manual contour	0.73%	Yes	0.994^2^ (0.987, 0.998)	Excellent
Semi-automated	Visual-based	Automated ellipsoid	0.77%	Yes	0.994^2^ (0.985, 0.997)	Excellent
Semi-automated	Visual-based	Automated contour	3.61%	No	0.995 (0.988, 0.998)	Excellent
Semi-automated	Rule-based	Automated ellipsoid	0.68%	Yes	0.995^2^ (0.988, 0.998)	Excellent
Semi-automated	Rule-based	Automated contour	3.55%	No	0.996 (0.991, 0.998)	Excellent
Fully-automated	Atlas-based	Automated ellipsoid	0.55%	Yes	0.997^2^ (0.993, 0.999)	Excellent
Fully-automated	Atlas-based	Automated contour	3.42%	No	0.996 (0.991, 0.998)	Excellent
Fully-automated^3^	nn-based	Automated ellipsoid	0.75%	Yes	0.996^2^ (0.990, 0.998)	Excellent
Fully-automated^3^	nn-based	Automated contour	4.21%	No	0.996 (0.990, 0.998)	Excellent

ICC, intraclass correlation coefficient; nn, neural network; r-TEM, relative technical error of measurement.

^1^Interpretion is based on the threshold of 1.5% reported by Perini et al. (2026).

^2^There is evidence of singularity due to potential overfitting. Results should be interpreted with caution.

^3^Seven measurements were excluded from the nn-based approach because they were spurious.

**TABLE 3:** Accuracy (r-TEM) of different measurement methods with the tape grouped by age.

Automation type	Measurement plane selection	Circumference type	Age ≤ 4.5 years (*n* = 13)		Age > 4.5 years (*n* = 10)	
			Value	Acceptable?^1^	Value	Acceptable?^1^
Manual	Visual-based	Manual ellipsoid	1.02%	Yes	0.75%	Yes
Manual	Visual-based	Manual contour	0.78%	Yes	0.76%	Yes
Manual	Rule-based	Manual ellipsoid	1.25%	Yes	0.95%	Yes
Manual	Rule-based	Manual contour	0.74%	Yes	0.72%	Yes
Semi-automated	Visual-based	Automated ellipsoid	0.66%	Yes	0.87%	Yes
Semi-automated	Visual-based	Automated contour	3.62%	No	3.58%	No
Semi-automated	Rule-based	Automated ellipsoid	0.69%	Yes	0.67%	Yes
Semi-automated	Rule-based	Automated contour	3.66%	No	3.42%	No
Fully-automated	Atlas-based	Automated ellipsoid	0.68%	Yes	0.36%	Yes
Fully-automated	Atlas-based	Automated contour	3.58%	No	3.24%	No
Fully-automated^2^	nn-based	Automated ellipsoid	0.87%	Yes	0.43%	Yes
Fully-automated^2^	nn-based	Automated contour	4.19%	No	4.22%	No

nn, neural network; r-TEM, relative technical error of measurement.

^1^Interpretion is based on the threshold of 1.5% reported by Perini et al. (2026).

^2^Seven measurements were excluded from the nn-based approach because they were spurious.

The two-way mixed effects ANOVA revealed that all different measurement methods showed excellent consistency with the tape measurements, with ICC ranging from 0.993 to 0.997 (Table 2). However, there was evidence of singularity due to potential overfitting for manual contour and automated ellipsoid; hence, these results should be interpreted with great caution. Furthermore, seven measurements were excluded from the nn-based approach for being spurious, resulting in slightly wide 95% confidence intervals.

**FIGURE 2:**
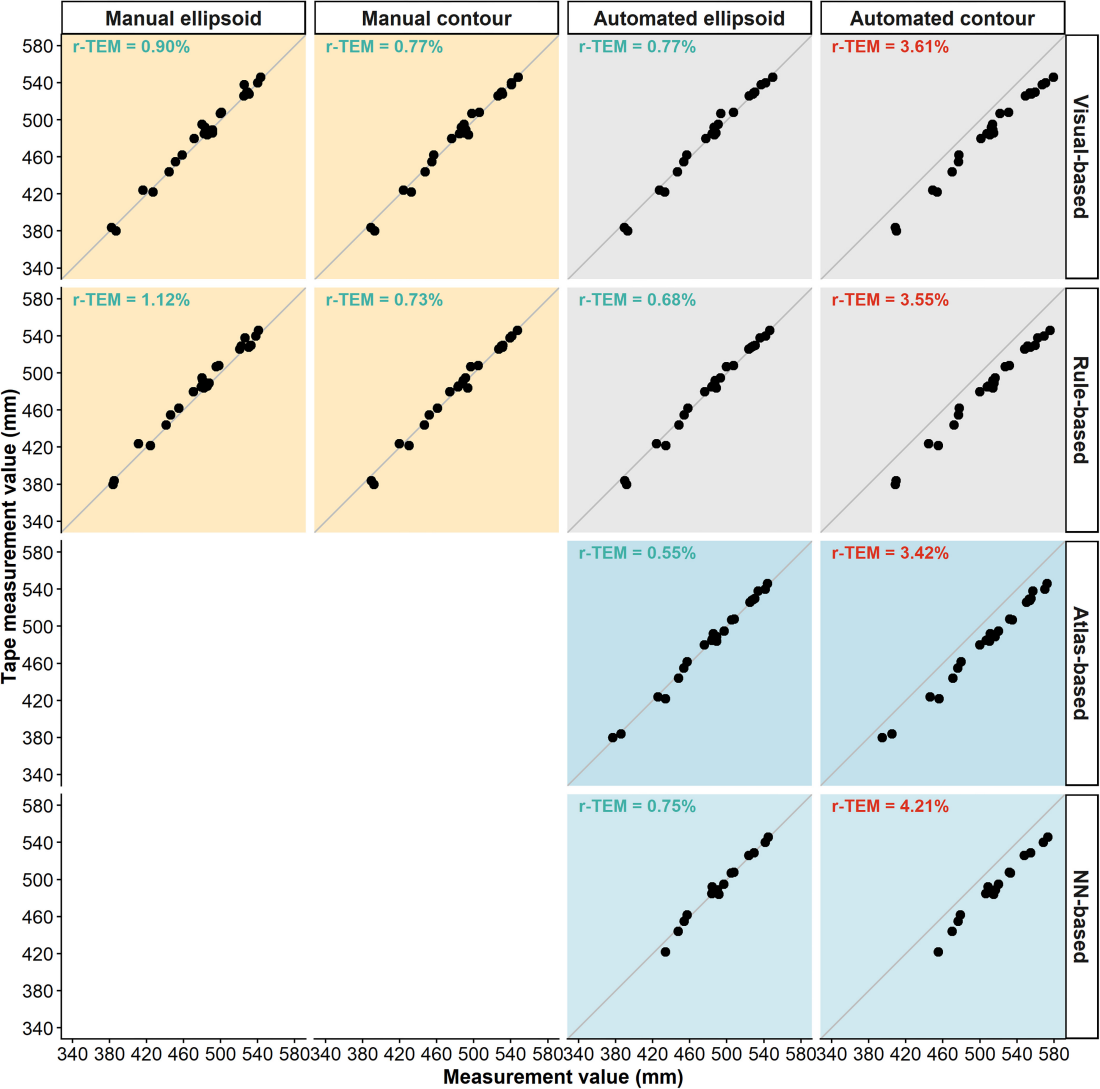
The scatterplots are divided into three panels, separating the manual (yellow background) from the semi-automated (gray background) and fully automated approaches (blue background), as well as determining the axial measurement plane (visual-based, rule-based, atlas-based, and nn-based) and the method for performing the measurement (ellipsoid and contour). The r-TEM values are presented at the top left of each plot, with green and red colors indicating acceptable and unacceptable accuracy with the tape measurements, respectively, based on the thresholds of Perini et al. (2026). A smaller deviation from the diagonal line indicates a strong linear association between the compared methods. Results are based on 23 children.

Figure 2 illustrates a panel of scatterplots comparing tape measurements with those from the different measurement methods. Overall, there was an almost perfect linear association between the tape measurement and the different manual, MRI-based measurements (visual- or rule-based plane selection with manual ellipsoid or contour circumference measurement), visual-based and rule-based with automated ellipsoid, as well as atlas-based and nn-based with automated ellipsoid, because the points overlapped perfectly with the diagonal line or were scattered at a small distance from the diagonal line. In contrast, the tape measurements yielded systematically lower values than visual-based and rule-based with automated contour, as well as atlas-based and nn-based with automated contour.

## Discussion

Measuring a child’s head circumference is an essential part of clinical pediatric examinations and serves as an important indicator of normal neurological development in early childhood. When interpreting pediatric cerebral MRI examinations, knowledge of head circumference development is also a helpful diagnostic parameter in the differential diagnosis of cerebral findings. This prospective study therefore evaluated the accuracy of different methods for measuring pediatric head circumferences using MRI scans. Various manual, semi-automated, and fully automated, MRI-based methods were used, and their reliability was validated against the gold standard of tape measurement.

Our results demonstrate that ellipsoid and contour measurements in visually defined optimal axial planes, as well as contour measurements with rule-based plane selection, provided high accuracy and excellent consistency with tape measurements. All head circumferences could be assessed correctly as age-appropriate normal or pathologic. There was no advantage of contour circumference measurements that mimick tape measurement or a standardized (rule-based) definition of the optimal measurement plane. In addition to reliable manual measurement techniques, the established fully automated, atlas-based algorithm also achieved an excellent consistency with the tape measurement. The fully automated, nn-based method also yielded good results in the evaluable data sets. However, in seven cases, the selection of the slice plane using this method was unsuccessful due to gross segmentation failures because the model had not been trained using data from young children. As these failures would have produced non-physiological circumferences and biased the comparison against the tape measure ground truth, we excluded these measurements from the results analysis, which also explains the reduced sample size and slightly wider confidence intervals (than those for atlas-based) for the nn-based approach. These cases involved very young children who had not yet completed myelination in T1, children with pronounced cerebral malformations, and children with postoperative changes. DeepBrainSeg, which was trained on adult data sets, thus reached its limits. The remaining 16 datasets yielded results that were highly comparable to the ground truth, so that after training DeepBrainSeg on pediatric MR datasets, comparably good results can be expected with this significantly faster, fully automated method. The measurements from the automated contour methods, in contrast to the automated ellipsoid, were on average larger than those by the tape measurement probably because contour measurement also captures concave sections of the head circumference that cannot be measured with a non-elastic tape.

The main strength of this study lies in its rigorous methodology and in comparing multiple measurement techniques in a real-world clinical setting. For good clinical practice, the different radiologists performing the tape and manual MRI-based measurements and establishing the algorithms were blinded to each other’s results. Instead of utilizing a retrospective design, as in prior studies (Rau et al., 2021; Mourão et al., 2023), we used prospective clinical measurements on the exact MRI date for validation, reducing the bias from incorrect or outdated clinical data and thereby increasing the accuracy of our results. Particularly in young children aged 6 years and younger, even minor time deviations could have significant impacts, as demonstrated by Bartholomeusz and Karns (2002). Our results for different manual, MRI-based measurement approaches (visual- versus rule-based plane selection, contour versus ellipsoid head circumference measurement) support the findings of prior studies; for example, Rau et al. (2021) demonstrated strong reliability and good agreement between MRI-based manual measurement and tape measurements when using elliptical ROIs for HC determination. Furthermore, our study extends these findings by including semi-automated and fully automated approaches, showing that the established fully automated, atlas-based algorithm provides reliable results with excellent consistency with the ground truth. We achieved an ICC of 0.997, whereas ICCs > 0.80 have already been considered excellent in other recently published fully automated neuroimaging algorithms (Barisano et al., 2025).

The excellent performance of our automated, atlas-based algorithm, and, in the evaluable cases, also of the neural network-based algorithm, is particularly significant, as both were developed and validated under realistic conditions. While Smith et al. (2013) excluded motion artifacts and skeletal deformities when establishing an automated measurement algorithm for CT images, we deliberately included MRI scans of pathologically sized heads, as well as complicating factors such as shunt devices and motion artifacts, which should enhance the generalizability of our findings. Like most automated brain segmentation models (Lenchik et al., 2019), our algorithm is based on a T1w 3D-MPRAGE sequence, which is widely available and used, thereby contributing to overall applicability. The absence of additional required sequences or complex post-processing tools is a significant advantage of our algorithm in its transition to clinical routine.

However, this study has some limitations, including the small sample size. The fully automated, nn-based approach was particularly affected by this, as an additional seven patients had to be excluded due to grossly incorrect segmentations, presumably because of their young age, pronounced cerebral malformations, postoperative intracranial changes, and the resulting anatomical differences, as DeepBrainSeg was developed for adults. Nevertheless, the neural network-based method, which is significantly faster than the atlas-based method, showed great promise, provided that the neural network is first trained on pediatric MRI datasets. Furthermore, the small sample size led to overfitting issues in some methods, compromising the credibility of the corresponding results. A larger cohort, as well as prior training on pediatric data sets, would strengthen the reliability of our results. The small calibration dataset also represents a limitation, as it does not reflect the wide anatomical variability in head shapes among children. Nevertheless, the atlas-based method demonstrated acceptable overall accuracy and excellent consistency in the metrics.

Additionally, while we aimed to mimic real-life conditions by including each pediatric patient with a manual head size measurement during the mentioned period, the number of pathologic head circumferences in our study population was 17.4%, including four microcephalic and three borderline head circumferences. Although we did not rely solely on MRI scans of a healthy sample, as in the previously mentioned studies (Vorperian et al., 2007; Smith et al., 2013), further investigations should also incorporate a higher proportion of pathological cases to improve validation, particularly within this subgroup.

## Conclusion

This pilot study demonstrates that measuring head circumference from 3D MPRage datasets using ellipsoid methods yields reliable results compared with the current gold-standard tape measurement in children; a visual definition of the optimal axial plane is sufficient. Furthermore, we developed a fully automated, atlas-based algorithm with automated ellipsoid measurements that showed excellent consistency with manual measurements from both MRI-based and clinical tape measurements. This automated tool could simplify head circumference measurement and provide a reproducible, reader-independent value, thereby improving the interpretation of neuroradiological findings. In addition, the innovative nn-based, fully automated approach would significantly accelerate the measurement process and appears to be very promising, provided that DeepBrainSeg is also trained beforehand on pediatric datasets. Further studies should be conducted to validate both fully automated approaches with larger sample sizes.

## Data Availability

The raw data supporting the conclusions of this article will be made available by the authors, without undue reservation.
